# Measurements of aerosol microphysical and chemical properties in the central Arctic atmosphere during MOSAiC

**DOI:** 10.1038/s41597-023-02586-1

**Published:** 2023-10-11

**Authors:** Benjamin Heutte, Nora Bergner, Ivo Beck, Hélène Angot, Lubna Dada, Lauriane L. J. Quéléver, Tiia Laurila, Matthew Boyer, Zoé Brasseur, Kaspar R. Daellenbach, Silvia Henning, Chongai Kuang, Markku Kulmala, Janne Lampilahti, Markus Lampimäki, Tuukka Petäjä, Matthew D. Shupe, Mikko Sipilä, Janek Uin, Tuija Jokinen, Julia Schmale

**Affiliations:** 1 Extreme Environments Research Laboratory, École Polytechnique Fédérale de Lausanne (EPFL) Valais Wallis, Sion, Switzerland; 2 Laboratory of Atmospheric Chemistry, Paul Scherrer Institute, 5232 Villigen, Switzerland; 3 Institute for Atmospheric and Earth System Research, INAR/Physics, Faculty of Science, University of Helsinki, 00014 Helsinki, Finland; 4 Leibniz Institute for Tropospheric Research, Permoserstrasse 15, 04138 Leipzig, Germany; 5 Environmental and Climate Sciences Department, Brookhaven National Laboratory, Upton, NY USA; 6 Cooperative Institute for Research in Environmental Sciences, University of Colorado, Boulder, CO USA; 7 National Oceanic and Atmospheric Administration, Physical Sciences Laboratory, Boulder, CO USA; 8 Climate and Atmosphere Research Centre (CARE-C), The Cyprus Institute, P.O. Box 27456, Nicosia, 1645 Cyprus; 9 Present Address: Univ. Grenoble Alpes, CNRS, INRAE, IRD, Grenoble INP, IGE, 38000 Grenoble, France

**Keywords:** Atmospheric chemistry, Atmospheric dynamics

## Abstract

The Arctic environment is transforming rapidly due to climate change. Aerosols’ abundance and physicochemical characteristics play a crucial, yet uncertain, role in these changes due to their influence on the surface energy budget through direct interaction with solar radiation and indirectly via cloud formation. Importantly, Arctic aerosol properties are also changing in response to climate change. Despite their importance, year-round measurements of their characteristics are sparse in the Arctic and often confined to lower latitudes at Arctic land-based stations and/or short high-latitude summertime campaigns. Here, we present unique aerosol microphysics and chemical composition datasets collected during the year-long Multidisciplinary drifting Observatory for the Study of Arctic Climate (MOSAiC) expedition, in the central Arctic. These datasets, which include aerosol particle number concentrations, size distributions, cloud condensation nuclei concentrations, fluorescent aerosol concentrations and properties, and aerosol bulk chemical composition (black carbon, sulfate, nitrate, ammonium, chloride, and organics) will serve to improve our understanding of high-Arctic aerosol processes, with relevance towards improved modelling of the future Arctic (and global) climate.

## Background & Summary

The Arctic atmosphere is experiencing considerable changes and is warming at a rate up to four times as fast as the rest of the world^1–3^. This phenomenon, referred to as Arctic amplification, has important consequences on the Arctic environment, e.g., more frequent, intense, and longer extreme events^4,5^ with impacts in mid-latitudes. Regionally, permafrost thawing has repercussions for land-based infrastructure and increases global methane^6^ and volatile organic compounds^7^ emissions, while sea ice decline triggers natural resource extraction plans, and social and economic challenges for Arctic indigenous people^8^. Among the various regional and global processes that contribute to the enhanced Arctic warming, warming through greenhouse gases forcing (e.g., carbon dioxide (CO_2_)), snow and ice-albedo feedbacks^9,10^, temperature feedbacks^11^ (Planck and lapse rate feedbacks), and ocean warming have often been identified as the key drivers of Arctic amplification^11,12^. Aerosols are a major component in the Arctic’s radiative balance^13–16^ and can be from local or remote sources, primary or secondary in origin, and anthropogenically (e.g., industrial activities, traffic and agriculture) or naturally-sourced (e.g., wind-blown dust or snow, sea-spray, and wildfires)^16,17^. Aerosols can directly affect radiative transfer by absorbing or scattering incoming radiation (aerosol-radiation interaction, ARI) or indirectly, by modulating cloud radiative properties through aerosol-cloud interactions (ACIs). However, ACI remains poorly understood and contributes to the largest uncertainty in radiative forcing estimates^18,19^, owing to a lack of observational evidence to evaluate models and to understand the sources of aerosols that contribute to cloud and fog formation. This lack of observations is particularly true in the central Arctic Ocean due to the complexity of monitoring *in situ* atmospheric variables in this remote location.

Most of the present-day knowledge on aerosol processes and seasonality in the Arctic^13,14,20,21^ has been gained from permanent land-based monitoring stations around the Arctic, e.g., refs. ^17,22^ or from short high-latitude aircraft, e.g., refs. ^21,23,24^ or ship-based campaigns, e.g., refs. ^25–29^, predominantly during summertime. With regard to the summertime bias, it is long known that it cannot represent the full annual aerosol cycle^13,14,18,22^. More recent results also suggest that land-based observations cannot fully represent the central Arctic Ocean^30^. Local processes or short regional extreme events may not always be captured at Arctic land-based stations^31^, highlighting the need for an investigation of more local processes and the local impact of aerosols on the radiative balance through ARI and ACI. The overall scarcity of aerosol observations in the central Arctic Ocean, especially during the dark winter and early spring time, was a key motivation for the international collaboration which led to the “Multidisciplinary drifting Observatory for the Study of Arctic Climate” (MOSAiC) expedition to take place in the central Arctic from October 2019 to September 2020. For an entire year, the Research Vessel (RV) *Polarstern*^32^ drifted with the central Arctic sea ice, hosting an extensive suite of experiments designed to study the coupled atmosphere-ice-ocean-ecosystem processes in one of the most climate-sensitive environments on the planet^33^. In particular, a vast ensemble of high time-resolution instruments for *in situ* monitoring of the central Arctic aerosols’ abundance and physicochemical characteristics was deployed. From the perspective of improving large-scale numerical models and future climate projections, the expedition provided a large dataset of climate-relevant variables through an entire year, for current and future generations of researchers to build upon. The combination of high time-resolution and year-round continuous measurements will allow for the drivers of seasonal variations to be unravelled, from the build-up of the anthropogenically-driven Arctic haze in winter and spring^34–36^, through the prevalence of naturally-sourced ultrafine particles in summertime^29,30,37^, to the more pristine autumn season, closing the annual cycle.

In this manuscript, we present an unprecedented year-round dataset of aerosol microphysics and chemical composition measurements performed in the central Arctic Ocean in the *Swiss container* during the MOSAiC expedition. These include measurements of aerosol particle number concentrations, size distributions, cloud condensation nuclei concentrations, fluorescent aerosol concentrations and properties, as well as aerosol bulk chemical composition and mass concentration (black carbon, sulfate, nitrate, ammonium, chloride and organics). We present an evaluation of the datasets’ quality, inferred from closure analysis between measured and derived variables and from intercomparisons between several of our measurements with redundant ones that were acquired with a close-by and independent instrument suite.

## Methods

### Instrumental setup

On September 20^th^ 2019, RV *Polarstern* left Tromsø harbour, Norway, towards the central Arctic Ocean to make year-long measurements, while trapped in the sea ice. The drift began on October 4^th^ 2019, when a suitable ice floe, to which *Polarstern* could be moored, was found. Apart from a detachment from the original ice floe between May 16^th^ and June 19^th^ 2020, for a logistical round-trip to Svalbard, *Polarstern* drifted in the central Arctic Ocean sea ice until July 31^st^ 2020, when the original ice floe disintegrated. On August 21^st^ 2020, RV *Polarstern* was brought to a new ice floe close to the North Pole and drifted again until September 20^th^ 2020. The detailed expedition track, along with the description of all atmospheric measurements performed onboard RV *Polarstern*, can be found in Shupe *et al*.^33^.

**Fig. 1:**
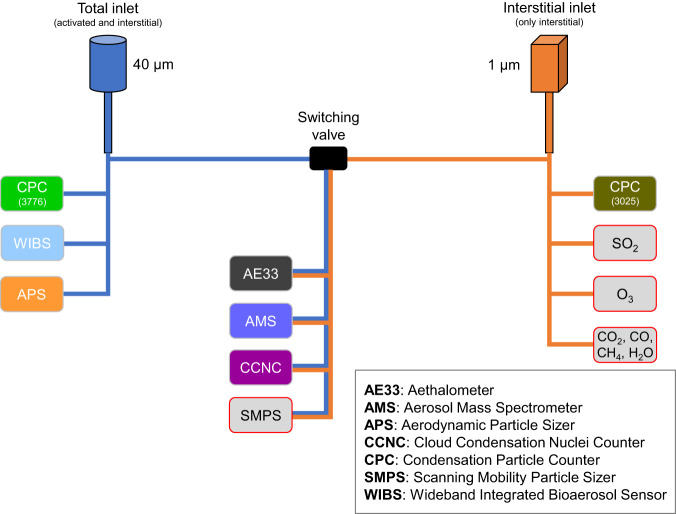
Schematic of the instrumental setup inside the Swiss container (adapted from Dada *et al*.^31^ and Beck *et al*.^68^). Aerosol particles were sampled from two distinct inlets located three meters apart: a total inlet which sampled all particles and hydrometeors up to 40 μm in diameter, and an interstitial inlet equipped with a 1 μm cyclone which sampled aerosols that did not activate as droplets in fog or cloud. The color code for each instrument is arbitrary and only used to refer to each instrument in Fig. 2. Instruments that are not described in the current paper are indicated with a grey box with red contours. A detailed description of trace gases measurements (sulfur dioxide (SO_2_), ozone (O_3_), carbon dioxide (CO_2_), carbon monoxide (CO), and methane (CH_4_)) is available in Angot *et al*.^41^. SMPS data are not discussed further due to instrumental issues during the expedition. We recommend the use of the ARM SMPS observations instead.

**Fig. 2:**
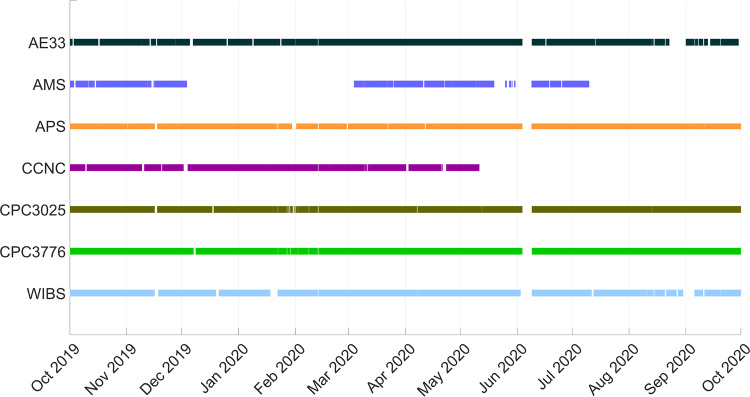
Summary of data availability for each instrument during the MOSAiC year. Periods with no measurements can arise from maintenance, instrumental failures, or from the presence of Polarstern in sovereign maritime zones (e.g., within Svalbard’s 12 nautical miles zone in early June). The longer gaps in the AMS and CCNC time series are due to instrumental failures.

Scientific activities during the expedition were carried out both on sea ice (through an instrument network spanning up to 50 km around RV *Polarstern*) and on the ship itself. The *Swiss container* was located on the D-deck of RV *Polarstern*’s bow, for which a schematic of the instrumental and inlet setup is provided in Fig. 1. The container was equipped with three inlets: 1) a total inlet with a flow of >15 L/min and an upper particle cutoff size of 40 μm for sampling particles and hydrometeors, 2) an interstitial inlet with a flow of >17 L/min and equipped with a 1 μm cyclone for sampling interstitial particles only, and 3) a new particle formation (NPF) inlet (not shown in Fig. 1 and not further discussed in the current manuscript). The inlets pointed upwards outside the container, with a length of 1.5 m, for a total approximate height of 15 m above sea level. The total and interstitial inlets were located 3 m apart and connected inside the container to a valve that switched hourly between the two inlets. This allowed for quantification of both activated and non-activated particles in case of foggy conditions for a targeted set of instruments. This design was specifically conceived to study aerosol in-cloud/fog processes, and enables measurements to infer the aerosol sources that are linked with activated and non-activated particles^38^. Inside the container, the temperature was kept constant around 20 °C and the relative humidity inside the inlets was maintained well below 40% with a heating system following the Global Atmosphere Watch (GAW) standards for aerosol sampling^39^. Temperature and relative humidity were measured inside bypasses to both inlets with two hygrometers model HC2 (Rotronic AG, Bassersdorf, Switzerland). The recorded time series, averaged to 10 min time resolution from the 5 min native one, were made available on PANGAEA repository (see “Data Records” section). The position of the switching valve (i.e., “interstitial” or “total”) during the campaign is also included in the aforementioned datasets. Summaries of the various measurements and available datasets considered in the current manuscript are provided in Table 1 and Fig. 2, respectively.

**Table 1:** List of measurements performed with the various instruments discussed in this paper. All of the measurements were continuously operated in the Swiss container, obtaining real-time data.

Measurement	Instrument	Size range	Native time resolution
Bulk size-resolved chemical composition and mass concentration of non-refractory submicron aerosols	High-Resolution Time-of-Flight Aerosol Mass Spectrometer (HR-ToF-AMS, Aerodyne Research, Inc.)	<1 µm (vacuum aerodynamic diameter, *d*_*va*_)	~90 s
Bulk equivalent black carbon mass concentration	Aethalometer (model AE33, Magee Scientific)	Behind switching valve, hence depending on inlet	1 s
Coarse mode aerosol size distributions	Aerodynamic Particle Sizer (APS model 3321, TSI)	500 nm - 20 µm (aerodynamic diameter, *d*_*a*_)	20 s
Size-resolved number concentrations and fluorescence of aerosols	Wideband Integrated Bioaerosol Sensor (WIBS NEO, DMT)	500 nm - 20 µm (optical diameter, *d*_*opt*_)	125 Hz single-particle counter
CCN number concentrations at various supersaturation levels	Cloud Condensation Nuclei Counter (CCNC model CCN-100, DMT)	Behind switching valve, hence depending on inlet	1 s
Aerosol number concentrations	Condensation Particle Counter (CPC, model 3025 and 3776, TSI)	CPC 3025 (interstitial inlet): 3 nm -<1 µm CPC 3776 (total inlet): 2.5 nm - 10 µm (*d*_*opt*_)	10 s

Additional instruments from another laboratory container were used for intercomparisons, mass closure analysis, and corrections, including an additional Cloud Condensation Nuclei Counter (CCNC), a Scanning Mobility Particle Sizer (SMPS), and an Ultra-High-Sensitivity Aerosol Spectrometer (UHSAS). These instruments were located in the Aerosol Observing System (AOS) container, operated as part of the United States Department of Energy Atmospheric Radiation Measurement (ARM) user facility^40^ during MOSAiC. The ARM container was also located on the D-deck of *Polarstern*’s bow about 1.5 m away from the *Swiss container*, with a total aerosol inlet of 5 m in length reaching around 18 m above sea level. These datasets are available freely online and a link to access them is provided in the “Data Records” section. More information on the ARM container, its operation and instrumental setup can be found in Uin *et al*.^40^, Boyer *et al*.^30^, and Angot *et al*.^41^.

### Inlet losses characterization

The inlet system presented in Fig. 1 was characterized for losses using a particle loss calculator (PLC)^42^. The overall sampling efficiency is approximated in the model by solving equations for sedimentation, diffusion, turbulent inertial deposition, inertial deposition in sampling line bends, contractions and enlargements or for electrostatic deposition, interception, and coagulation processes^42^. The aforementioned quantities depend on the inlet system considered (e.g., flow rate, tube length and diameter, inclination angles and curvatures) and on the particle size, such that smaller particles will be more sensitive to diffusion losses through Brownian motion and larger particles to sedimentation losses through gravitational settling. For the calculations, we assumed an averaged particle density of 1.5 g/cm^3^, representative of the Arctic^43–45^. For each individual pathway leading to the different instruments, with the exception of the AMS, the particle losses inside the sampling lines range from ~30 to ~50% for particles smaller than 5 nm (where only the two CPCs can measure such small particles), from ~10 to ~30% for particles between 5 and 10 nm, from ~0.5 to ~15% for particles between 10 and 100 nm, and from nearly 0 to ~3% for particles between 100 nm and 1000 nm. The AMS was operated at a lower flow rate than the other instruments (0.07 L/min, see “Aerosol chemical composition and mass concentration” section) and the calculated losses for the corresponding sampling lines are consequently higher. In the size range relevant for the AMS (i.e. ~50–1000 nm), the calculated inlet losses are below 10% between 50 and 700 nm and up to 20% between 700 and 1000 nm. In summary, for submicron particles (i.e., 10 to 1000 nm) losses are hence very small (0–10%), in line with losses reported for similar inlet systems deployed at Arctic ground-based stations^46^, and are not further accounted for.

In the supermicron size range, for the sampling line leading to the CPC3776, we calculated losses between 3 and 20% within the size range from 1 to 3 µm, and larger than 40% above 4 µm. Finally, for the sampling lines leading to the APS and WIBS, calculated losses vary from nearly 0 to 4% for particles smaller than 3 µm, from 4 to 10% for particles between 3 and 5 µm, between 10 and 35% for particles between 5 and 10 µm, and more than 35% for particles larger than 10 µm. Hence, reported supermicron aerosol number concentrations represent a lower estimate.

### Equivalent black carbon mass concentration

#### Instrument description and in situ operation

Measurements of equivalent black carbon (eBC) were performed using a commercial aethalometer model AE33 (Magee Scientific, Berkeley, USA). The instrument was connected to the switching valve (Fig. 1), with a sample flow of 2 L/min, biweekly verified. The dual-spot technology of the instrument ensures a real-time compensation of what is referred to as the loading effect^47^. In practice, the aerosols are collected on a filter and the attenuation of transmitted light is measured at 7 different wavelengths (370, 470, 520, 590, 660, 880 and 950 nm) with a time resolution of 1 s. The instrument reports the measurements as eBC mass concentrations, as inferred from the measured attenuation at the aforementioned wavelengths, using Eq. 16 in Drinovec *et al*.^47^. The data obtained from measurements at 880 nm were used for computing and reporting eBC mass concentrations, using the corresponding standard mass absorption cross-section (MAC) value of 7.77 m^2^g^−1 ^^47^. Measurements at all wavelengths (λ) were used to report the aerosol optical absorption coefficients, obtained by multiplying the eBC(λ) mass concentrations by the default MAC(λ) values of 18.47, 14.54, 13.14, 11.58, 10.35, 7.77 and 7.19 m^2^g^−1^, for the wavelengths 370, 470, 520, 590, 660, 880, and 950 nm, respectively. These can be used for source apportionment or for the computation of the Absorption Ångström Exponent (AAE)^48^. The instrument’s manufacturer does not report specific values for measurement uncertainties, but a recent study intercomparing multiple AE33 aethalometers estimated that noise (which is not the only source of measurement uncertainty) accounted on average for 10% of the averaged ambient eBC mass concentration^49^. Noise was defined as one standard deviation of the eBC mass concentration of dry filtered air. The authors reported average noise levels of 31 ng/m^3^ for eBC measurements at 880 nm and at 1 min time resolution. At 10 min time resolution, this noise level can be estimated to be 9.8 ng/m^3^ (31/√(10) ≈ 9.8, using Eq. 4 in Fröhlich *et al*.^50^). Time-averaging will in this case only lower the electronic noise but not the uncertainties associated with the measurement technique^51^. We further evaluated the need for a loading effect correction, in addition to the dual-spot compensation, by inspection of the concentration during filter tape changing periods and found that no additional correction was needed.

#### Data processing and cleaning

As time integration reduces noise (see above), the raw 1 s dataset was first averaged to a time resolution of 1 min. Then, outliers of more than 3 times the median absolute deviation (MAD) from an hourly moving window were removed. This commonly used median-based method for excluding outliers relies on the assumption that the data are normally distributed and then identifies extreme values on both sides of the distribution. A visual inspection of the resulting time series after applying the MAD method confirmed that no “true” signal (i.e., representative of ambient conditions) was removed in the process. After correcting for artefacts that occurred when switching between the total and interstitial inlets, which caused a difference pattern of mean and standard deviation of the measurements between odd and even hours (see the “Inlet switching correction” section, likewise for the two CPC, AMS, and CCNC datasets), the data were finally averaged to a 10 min time resolution. Based on a visual inspection of the entire dataset, we further excluded periods of strong noise and intense negative spikes. These data points may have emerged from the averaging of the initially noisy 1 s time resolution dataset and/or from the dual-spot compensation, which may lead to the presence of a strong negative outlier right after a positive one.

### Aerosol chemical composition and mass concentration

#### Instrument description, in situ operation, and processing of raw data

The bulk size-resolved chemical composition and mass concentration of non-refractory aerosols smaller than 1 µm (NR-PM_1_) in vacuum aerodynamic diameter (*d*_*va*_), was measured using a High-Resolution Time-of-Flight Aerosol Mass Spectrometer (HR-ToF-AMS, Aerodyne Research, Inc.), for which detailed description, functioning principles and field deployment procedures have been extensively described in the literature^52,53^. In short, during MOSAiC, ambient air was sampled alternatively every hour from the total and interstitial inlets into an aerodynamic lens with a 1 µm critical orifice and a flow of 0.07 L/min. The particle size was then determined based on the particle time-of-flight (PToF) across a fixed distance in the instrument’s sizing region, under vacuum (~10^−5 ^Torr). The vaporizer, consisting of a resistively heated tungsten surface, was set to a temperature of 600 °C (operating current ~1.25 A), verified in June 2019 with a heater calibration using size selected 225 nm sodium nitrate (NaNO_3_) particles^54^. In theory, with a vaporizer temperature of 600 °C, only non-refractory (NR) species (here defined as the species that are flash-vaporized at temperatures below or equal to 600 °C) are measured, such as sulfate (SO_4_^2−^), nitrate (NO_3_^−^), ammonium (NH_4_^+^), chloride (Cl^−^) and some organic matter (Org). Thus, refractory materials such as sea salt, black carbon, crustal material or metal oxides are mainly excluded^55^. It has, however, been shown that with a proper tuning and calibration of the instrument, sea salt could be detected and quantified, albeit with considerable uncertainties^56,57^. In the time-of-flight mass spectrometer (ToF-MS), where ions are separated based on their mass-to-charge (*m/z*) ratio, two operational modes defined by the ion path length are available: the V-mode and the W-mode^52^. The W mode is characterized by a longer ion flight path, hence a higher spectral resolution but decreased signal intensity due to ion losses along the path. Since the Arctic has very low aerosol concentrations, even during haze conditions, only the V-mode was operated during the expedition, to maximize the signal and to lower the detection limits (reported in Table 2). Furthermore, the instrument was operated sequentially in the “mass spectrum (MS) mode” and “PToF mode” with an effective time resolution of about 90 s. Due to turbo pump failures, the AMS was not running between December 5^th^ 2019 and February 29^th^ 2020, between May 30^th^ 2020 and June 6^th^ 2020, and was completely shut down after July 10^th^ 2020.

**Table 2:** Calibration-derived relative ionization efficiencies for ammonium and sulfate (RIE_NH4_ and RIE_SO4,_ respectively), and detection limits for the 5 main aerosol species at 90 s time resolution, for the three periods of Oct-Dec, Mar-May, and Jun-Jul. The detection limits were calculated as three times the standard deviation of the species mass concentration during blank filter (HEPA filter) measurements and are valid for a time resolution of 90 s (native). Organics have larger detection limits, associated with a lower signal-to-noise ratio, as it includes ion fragments at higher m/z (especially in Jun-Jul, the Organics signal is very noisy during filter period measurements).

	RIE_NH4 [–]_	RIE_SO4 [–]_	Detection limits for sulfate, nitrate, ammonium, chloride, and organics, respectively [µg/m^3^]
**Oct-Dec**	3.3	1.15	0.017, 0.011, 0.001, 0.055, 0.284
**Mar-May**	3.4	1.3	0.102, 0.069, 0.027, 0.055, 0.718
**Jun-Jul**	3.4	1.3	0.084, 0.152, 0.261, 0.071, 1.029

The AMS data were analyzed and processed using SQUIRREL (SeQUential Igor data RetRiEvaL) v1.65B and PIKA (Peak Integration by Key Analysis) v1.25B^58^ within the IGOR Pro v9.00 software (Wavemetrics, Inc., Lake Oswego, OR, USA). This was done separately for the three different periods of available measurements, Oct-Dec, Mar-May, and Jun-Jul, as the instrument was each time in a different state (after long down times). The ionization efficiency of nitrate (*IE*_*NO*3_), and relative ionization efficiencies of ammonium and sulfate (*RIE*_*NH*4_ and *RIE*_*SO*4_, respectively) were determined (see Table 2) using standardized mass balance methods with regular on-site calibrations using monodisperse, number concentration-defined, ammonium nitrate (NH_4_NO_3_), and ammonium sulfate ((NH_4_)_2_SO_4_) particles^55,59,60^. *IE*_*NO*3_ corresponds to the total number of ions detected per number of molecules vaporized and is used as a calibration factor to calculate the mass concentration of the different species in nitrate equivalent, while relative ionization efficiencies (such as *RIE*_*NH*4_ and *RIE*_*SO*4_) are used to get the species’ absolute mass concentration from the nitrate equivalent one. For the Oct-Dec period, *IE*_*NO*3_ and *RIE*_*NH*4_ values were obtained from averaging the values derived for all calibration procedures during the period (SD(*IE*_*NO*3_/airbeam) = 5.54 * 10^−13^, SD(*RIE*_*NH*4_) = 0.025), while a single calibration was used to retrieve *RIE*_*SO*4_. Similarly, *IE*_*NO*3_ and the *RIE* values for the Mar-May period were obtained from a single calibration on April 12^th^. For the Jun-Jul period, the calibrations failed to produce usable results due to instrumental turbo pump failures and the values from the Mar-May period were used for both *RIE*_*NH*4_ and *RIE*_*SO*4_. Within PIKA, the fragmentation table was adapted for air fragmentation patterns using periods of zero measurements, performed on a regular basis (several times per month) using High-Efficiency Particulate Absorbing (HEPA) filters. In particular, the abundance of isotopic nitrogen 15 (^15^N_2_^+^) at *m/z* 29 was adjusted by constraining ^15^N_2_^+^ to N_2_^+^ in order to determine CHO^+^ abundance, since CHO^+^ and ^15^N_2_^+^ are less than 0.001 *m/z* apart. In V-mode, the deployed HR-ToF-AMS has a mass resolving power of about 2100 (m/Δm)^52^ so that, at *m/z* 29, we can reliably separate ions at 0.014 *m/z* apart (29/2100 = 0.014). The fractional amount of CO_2_^+^ in the gas phase was adjusted similarly by determining the relation between CO_2_^+^ (*m/z* 44) and C_2_H_3_O^+^ (*m/z* 43) during filter periods. A time-dependent airbeam correction factor was applied to the dataset (medians of 1.23, 0.99, and 0.93 for the Oct-Dec, Mar-May, and Jun-Jul periods, respectively), along with a time and composition-dependent collection efficiency (CDCE)^61^, accounting for particles bouncing of the heater.

Regarding measurement uncertainties, there is no strict consensus for their quantification, as they depend on a large variety of factors (e.g., the *IE*_*NO*__3_ estimation, collection efficiency (CE) estimation, and chemical composition)^56^. Bahreini *et al*.^62^ estimated the propagated, overall uncertainty for the total AMS mass concentration to 20–35%, where the uncertainty in CE estimation and *RIE*s of the different species are the major contributors to the overall uncertainty^61^. A statistical ion counting error can however be computed, assuming that the probable distribution of counted ions can be modelled as a Poisson distribution^60^. Summing the errors for all *m/z* that contribute to each species (i.e. SO_4_^2−^, NO_3_^−^, NH_4_^+^, Cl^−^, and Org), we get median error contributions to the measured mass concentrations of 1.99, 34.35, 20.22, 43.29 and 9.73% for SO_4_^2−^, NO_3_^−^, NH_4_^+^, Cl^−^, and Org, respectively, for the Mar-May period. Hence, depending on the considered species, these can represent a small or substantial part of the overall measurement uncertainty mentioned above.

#### Data filtering and switching correction

The following periods were removed from the final dataset: when the airbeam correction factor was larger than 2 or smaller than 0, outliers (defined as more than 3 times the standard deviation of half an hour moving average), all calibrations, filter periods and data non-representative of ambient conditions (e.g., electronic interferences at the proximity of a cellphone or any period with a disconnection of the AMS inlet from the main ambient line for diverse reasons).

#### Scaling factor from mass closure with the ARM SMPS

**Fig. 3:**
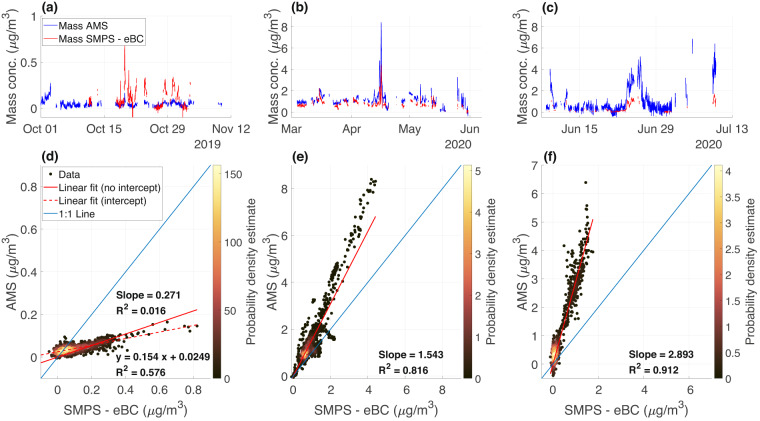
Mass closure between the 10-min averaged AMS and the SMPS (from which eBC is subtracted). The 10-min averaged time series for the mass concentration measured with the AMS and SMPS (- eBC) are shown in panels (**a**–**c**) for the three periods Oct-Dec, Mar-May and Jun-Jul, respectively. The corresponding density scatter plots with linear fit are shown in panels (**d**–**f**). The color bars indicate the probability density function of the distributions, calculated with a kernel density estimate (large values indicate close proximity and large density of data points). The low R^2^ between the AMS and SMPS (- eBC) mass for the Oct-Dec period (R^2^ = 0.016) is due to the forcing of the linear fit through the origin (R^2^ = 0.576 when shifting the slope to y = 0.154x + 0.0249, corresponding to the dashed red line in the figure in panel (**d**)).

A mass closure analysis between the AMS and ARM SMPS was performed independently for the three periods Oct-Dec, Mar-May and Jun-Jul, and yielded the following slopes: 0.271 (R^2^ = 0.016), 1.543 (R^2^ = 0.816) and 2.893 (R^2^ = 0.912) for the three respective periods. For the Oct-Dec period, the R^2^ value is very low (0.016) as a result of the forcing of the linear regression through the origin. This value, which represents the statistical goodness of the fit (not to be confounded with a measure of the correlation), changes to 0.576 if the linear regression is not forced through zero. In other words, the measurements from the two instruments during this period are not randomly distributed in a point cloud but are well correlated (Pearson’s correlation ρ = 0.759), despite the low R^2^ value reported (see Fig. 3). The resulting slopes mentioned above can be used as scaling factors ( = 1/slope) on the AMS data (see “Usage Notes” section). A detailed description of the mass closure analysis and resulting comparison is provided in the “Technical Validation” section.

### Coarse mode aerosol size distribution

#### Instrument description

The TSI 3321 Aerodynamic Particle Sizer Spectrometer (APS) measured aerosol number size distributions between 0.5 and 20 μm (aerodynamic diameter) in 52 bins, with a total (sheath + aerosol sample) flow of 4.5 L/min. The measurement principle is based on the acceleration of particles in response to the accelerated sample flow, and the particle time of flight between two laser beams is converted to an aerodynamic diameter, as larger particles have higher inertia and thus accelerate more slowly. The manufacturer reports a +/− 10% measurement uncertainty for the model 3321, as one could expect from the common 10% counting error associated with optical counting of particles^63^.

#### Data processing and correction

The data were processed using Python and the 20 s raw data were averaged to 1 min intervals. Time periods with zero filter measurements (using HEPA filters), which served to verify the instrument’s proper functioning, and periods with unstable flow that affected number concentrations were removed. As the APS had some flow rate irregularities during the campaign, we compared the size distribution against the WIBS and UHSAS (operated in the ARM container) and a correction factor for the APS size distributions was applied per bin based on the comparison of WIBS and APS number size distributions. This process, and the resulting comparison, are further discussed in the “Technical Validation” section.

### Fluorescent aerosol measurements

#### Instrument description

The Wideband Integrated Bioaerosol Sensor – New Electronics Option (WIBS NEO; Droplet Measurement Technologies, Longmont, CO, USA) is designed to measure fluorescent aerosols. It measures the size, asymmetry and fluorescence of particles with an optical diameter of 0.5 to 20 µm. The instrument uses a laser at 635 nm wavelength to detect single particles. Detected particles are excited by two ultraviolet (UV) xenon flashlamps at wavelengths of 280 and 370 nm and their emitted light is measured by two photomultipliers with bandwidths of 310–400 nm, and 420–650 nm. The WIBS counts excited particles at a maximum frequency of 125 Hz, which corresponds to a maximum concentration of 2.5*10^4^ particles/L with a sample flow of 0.3 L/min. The manufacturer does not report a specific measurement uncertainty, but the counting error from the optical counter is about 10%^63^. Measurements of the background intensity were performed automatically every 26 h for 5 min by firing the UV flashlamps in the absence of any particles. Weekly zero measurements were performed with HEPA filters and the sample flow was verified weekly.

#### Data processing

The data were processed for each month separately with the WIBS toolkit v1.36 for IGOR (Droplet Measurement Technologies). According to Savage *et al*.^64^ and Moallemi *et al*.^65^, excited particles were classified as fluorescent if their fluorescent intensity exceeded the background intensity by three standard deviations (3σ) and as hyper-fluorescent if the fluorescent intensity exceeded the background intensity by 9σ. Excited particles with a lower fluorescent intensity were considered to be non-fluorescent. The combination of two excitation wavelengths and two emission detector wavebands allows the classification of fluorescent particles into seven types: A, B, C, AB, AC, BC, and ABC^66^. With a careful data analysis, these classification types can be used to understand the sources of the measured fluorescent aerosols. Averaged time series data at 1 h time resolution were created for the following parameters, for excited, fluorescent, and hyper-fluorescent particles: particle number size distribution and fluorescence. Zero measurements were removed from the final dataset.

### CCNC

#### Instrument description

The Cloud Condensation Nuclei Counter (CCNC), model CCN-100 from Droplet Measurement Technologies (DMT, Boulder, USA) consists of a cylindrical continuous-flow chamber in which aerosols are exposed to a defined, constant supersaturation. The supersaturation is generated by applying a temperature gradient at the column walls, and as diffusion of water vapor in air is faster than diffusion of heat^67^, the column centerline is supersaturated. Particles form droplets when they activate at a supersaturation lower than the set supersaturation and activated droplets are counted by an optical particle counter.

The measurements were performed in 1-h cycles, with a 0.5 L/min sample flow and an external 2 L/min make up flow, where the supersaturations 0.15, 0.2, 0.3, 0.5 and 1.0% were measured. The supersaturation of 0.15% is measured for 20 min, as it takes longer to equilibrate, and the remaining supersaturations were measured for 10 min each. The instrument was calibrated in July 2019 before the campaign, and in March and April 2020 during the campaign. Based on the inter-variability of the calculated supersaturation levels during these calibrations, we can expect values ranging from 0.15–0.20, 0.20–0.25, 0.29–0.33, 0.43–0.5, 0.78–1.0% for the nominal supersaturations of 0.15, 0.2, 0.3, 0.5 and 1.0%, respectively. The counting error for the CCNC is associated with the error in the optical counting of particles and is about 10%^46,63^.

#### Data processing

Data were removed during the cooling cycle (i.e., the time when the measurement cycle starts again and the temperature is cooled to set the lowest supersaturation), which corresponds roughly to the first 10 min of each hour (so 50% of the 0.15% supersaturation period). Also, the first minute of data after switching to the next supersaturation step were removed, to account for temperature stabilization in the instrument’s column. Data were finally averaged to 1 min time resolution (native one as 1 sec) for each supersaturation level separately.

### Total particle number concentration

#### Instruments

To measure the total particle number concentration behind both inlets, we used two condensation particle counters (CPCs), one model 3025 by TSI, Inc. (referred to as CPC3025) and one model 3776 by TSI, Inc. (referred to as CPC3776). We ran the CPC3025 behind the interstitial inlet and the CPC3776 behind the total inlet (see Fig. 1). The minimum detectable particle diameter (50% counting efficiency, *d*_*p50*_) for the CPC3025 (CPC3776) is 3 nm (2.5 nm) with a maximum detectable particle concentration of 9.99*10^4^ cm^−3^ (3*10^5 ^cm^−3^). For particle number concentration below the maximum threshold, the manufacturer reports a measurement’s uncertainty of +/− 10%. The sample air of both CPC’s (sample flow: 0.3 L/min for each) was taken directly from the inlet lines, when they entered the container and before it was distributed to other instruments, and the sample lines of both instruments had a length of 400 mm, for comparability. We performed weekly zero tests with HEPA filters on both CPCs.

#### Data processing

Both datasets were cleaned from calibration and zero-check filter periods. Additionally, data from the CPC3025 was used to create a pollution detection algorithm (PDA), which is described in Beck *et al*.^68^. The pollution flag derived from the PDA was used to clean other instruments’ data from primary pollution influences (see “Pollution detection” section).

### Inlet switching correction

We applied two corrections. First, the switching valve caused data distortion, observed at every full hour (i.e., when the valve turns and the ambient sampling changes from one inlet to another, there is a brief moment with underpressure in the inlet lines). Consequently, all data points within ± 2 min of the full hours were conservatively removed for the instruments located behind the switching valve (i.e., AE33, AMS, and CCNC).

Second, during some periods, we observed a difference pattern of mean and standard deviation of the measurements between even and odd hours, most probably caused by a persistent pressure drop in the inlet lines, resulting in a proportional reduction of the concentration measurements. For the two CPC datasets, we corrected for this artefact by calculating two correction factors, derived by dividing the median particle number concentrations of 3 minutes before (after) the start (end) of the affected period by the median particle number concentration of 3 minutes after (before) the start (end) of the affected period. The data points of the affected period (i.e. during interstitial inlet measurements) were then multiplied by the linearly interpolated correction factor at the corresponding timestamp^69^. The data points of the total inlet measurement period were chosen as the reference to represent the “true” values as they corresponded to the ambient levels (baseline) of surrounding unaffected periods. For the aethalometer, AMS, and CCNC measurements, negative values or near-zero concentrations prevented the use of this multiplication/division-based method (increased offset if the affected period has negative values or unphysical division by zero), so that an adapted addition/subtraction-based method was used. There, the 1-h arithmetic mean of interstitial inlet measurements and the mean of the two adjacent hours of total inlet measurements were subtracted, and the resulting difference was added as a constant to the data points of the interstitial inlet measurements. This information is provided in the metadata of the published datasets. Finally, such a difference pattern was not observed for the WIBS and APS datasets, so that no corrections were needed.

In the Arctic, significant concentration changes can happen under certain conditions, e.g., changing wind direction, changing transport pattern, and evolving boundary layer dynamics. However, the 1-h cyclicity at which we observe the differences discussed above, and the evident relation with the switching valve operation indicate that such variations are non-representative of the “true” Arctic aerosol loading baseline and need to be corrected for. Each affected period was visually inspected, before and after correction, to ensure that the “true” variability in the signal (e.g., smooth increase/decrease in concentration during the passing of an air mass) would not be affected by these corrections.

### Pollution detection

Atmospheric measurements of aerosols can be challenging in pristine locations such as the central Arctic due to disturbing emissions from local activities (e.g., exhaust by *Polarstern*’s engine and vents, skidoos, on-ice diesel generators^68^).

Thus, the aethalometer, WIBS, CCNC, and CPC3025 data were cleaned from the influence of fresh local pollution emissions using a pollution flag developed by Beck *et al*.^68^, where a multi-step pollution detection algorithm (PDA) was applied to the interstitial CPC dataset (CPC3025) at 1 min time resolution. This pollution flag identified 62% of all available data points, at 1 min time resolution, as being influenced by local pollution emissions. Spring and summer are most affected by local contamination while winter is least affected^68^. Additionally, for the WIBS data, data points with more than 10 polluted minutes within an hour were removed from the final dataset. For the aethalometer, this pollution flag was converted to the 10 min time resolution of the final dataset by setting a condition, where, if more than 1 data point is polluted in a 10 min moving window, the entire 10 min period is defined as polluted. An additional pollution flag, this time derived from applying the PDA to the CPC3776 dataset, was used to clean the CPC3776 time series. The following parameters were used in the PDA script to derive this pollution flag: power law filter with *a* = 0.35 cm^−3^s^−1^ and *m* = 0.58 s^−1^, and with upper and lower thresholds of 10^4 ^cm^−3^ and 60 cm^−3^, respectively. The neighbouring points filter was activated along with the median deviation filter with a median factor of 1.4. Finally, the sparse filter was also activated, with a window size of 30 data points and a sparse threshold of 6 data points. Overall, this pollution flag identified 69% of available measurements, at 1 min time resolution, as being polluted. Applying two different pollution flags for the two CPCs was needed as the two instruments may not have had exactly the same exposure to fresh local emissions from the ship’s stack due to the difference in the inlet locations and heights. This is partly seen in the different percentages of data points identified as polluted (62% when the PDA is applied to the CPC3025 dataset and 69% when applied to the CPC3776 dataset).

Since ship pollution observed on RV *Polarstern* peaked at a diameter of approximately 30 nm^68^, the APS dataset was less affected by pollution, also reflected in the smaller amount of pollution spikes compared to datasets including smaller diameters. Therefore, the PDA was applied to the APS total number concentration with the following parameters: interquartile range (IQR) filter with an IQR window of 2880 min and an IQR factor of 1.7. The upper threshold was set to 300 particles/cm^3^ and the neighbour decision was activated. Additionally, the median filter was applied with a median time window of 30 min and a median factor of 1.5, and the sparse filter, with a window size of 30 data points, and a sparse threshold of 20 data points. As a result, 16% of all available APS measurements, at 1 min time resolution, are defined as polluted.

Similarly, aerosols with sizes in which freshly emitted particles from the ship’s stack are found, are not efficiently measured by the AMS. This caused the CPC pollution mask to classify AMS data points as polluted, while they did not appear (visually and chemically) to be polluted. Consequently, a separate pollution mask was developed to remove freshly emitted pollution, following a cosine similarity approach described by Dada *et al*.^31^. In short, a minimum of two spectra, representative of fresh pollution, were chosen per month and averaged to create a reference spectrum of known pollution. We selected these spectra based on wind direction (possible influence of the ship’s emissions mainly between 120 and 240° from the stack), concentrations of ship emissions markers measured by the AMS (e.g., C_4_H_7_^+^, C_4_H_9_^+^ and C_6_H_7_^+^) and observations from other instruments consistent with fresh pollution (CPC and aethalometer). We then computed the similarity between this reference spectrum and each of the data points using the following formula (Eq. 1):

1 $$\begin{array}{c}\cos \theta =\frac{A\cdot B}{\left|\left|A\right|\right|\cdot \left|\left|B\right|\right|}=\frac{{\sum }_{i=1}^{n}{A}_{i}{B}_{i}}{\sqrt{{\sum }_{i=1}^{n}{A}_{i}^{2}}\cdot \sqrt{{\sum }_{i=1}^{n}{B}_{i}^{2}}}\end{array}$$

where *A* and *B* are the spectra at each data point and the reference polluted spectrum, respectively, and *A*_*i*_ and *B*_*i*_ are the components of these vectors (i.e. the fragments of the spectra). A threshold, above which data are considered polluted, was chosen at the *cos θ* value where 80% of the data points were outside of the 120–240° wind direction polluted window. We applied this method separately on three different periods, Oct-Dec, Mar-May and Jun-Jul and found thresholds of 0.41, 0.59 and 0.61, respectively. Additionally, a sparse filter with a moving window spanning 60 datapoints (approx. 1h30) was applied to define periods as entirely polluted, where more than 60% of the data points within the window were already classified as polluted by the cosine similarity method. With this method, 49% of the available AMS measurements, at 90 sec time resolution, are identified as being directly influenced by local pollution emissions.

## Data Records

Table 3 summarizes data records for the datasets described in this work, with links to the open access PANGAEA repository, where they can be freely downloaded in tab-delimited text format. Table 4 summarizes the list of attributes and respective definition for the various files archived on PANGAEA. All datasets contain the following common variables: “*Date/Time*” (date and time of measurements in UTC), “*Event*” (event list of MOSAiC campaign PS122), and “*Latitude*” and “*Longitude*” of RV *Polarstern* in degrees north and east respectively.

**Table 3:** Datasets’ availability on PANGAEA repository. Links to the pollution flags derived from applying the PDA^68^ to the CPC3025 and CPC3776 datasets are also given. The final time resolution of the data products stored on PANGAEA is provided and the asterisk indicates that the data have been time-averaged.

Data product (instrument)	Time resolution	PANGAEA repository
eBC mass concentration (AE33)	10 min *	Heutte *et al*.^82^
Aerosol optical absorption coefficients (AE33)	10 min *	Heutte *et al*.^83^
Chemical composition and mass concentration of non-refractory submicron aerosols (AMS)	90 sec	Heutte *et al*.^84^
Coarse mode aerosol size distribution (APS)	1 min *	Bergner *et al*.^85^
Fluorescent aerosol measurements (WIBS)	1 h *	Beck *et al*.^86^
CCN number concentrations (CCNC)	1 min *	Bergner *et al*.^87^
Aerosol number concentration (CPC3025)	10 sec	Beck *et al*.^69^
Aerosol number concentration (CPC3776)	10 sec	Beck *et al*.^88^
Temperature and relative humidity in bypass to the interstitial inlet (Hygrometer)	10 min *	Heutte *et al*.^89^
Temperature and relative humidity in bypass to the total inlet (Hygrometer)	10 min *	Heutte *et al*.^90^
Pollution flag (PDA applied on 1 min averaged CPC3025 dataset)	1 min *	Beck *et al*.^80^
Pollution flag (PDA applied on 1 min averaged CPC3776 dataset)	1 min *	Beck *et al*.^91^

**Table 4:** List of attributes for the files archived on PANGAEA.

Variable	Definition
**AE33, eBC mass concentration**	
eBC_[ng/m^3^]	Equivalent black carbon mass concentration in units of ng/m^3^.
Flag_pollution	Pollution flag (1 = polluted; 0 = not polluted).
**AE33, optical absorption at all wavelengths**	
b_abs_λ_[Mm^−1^]	Aerosol optical absorption coefficients at wavelengths 370, 470, 520, 590, 660, 880, and 950 nm, in units of Mm^−1^.
Flag_pollution	Pollution flag (1 = polluted; 0 = not polluted).
**AMS**	
SO_4_^2−^_[µg/m^3^], NO_3_^−^_[µg/m^3^], NH_4_^+^_[µg/m^3^], Chl_[µg/m^3^], Org_[µg/m^3^]	Mass concentration of non-refractory submicron sulfate, nitrate, ammonium, chloride, and organics aerosols, respectively, in units of µg/m^3^.
Flag_pollution	Pollution flag (1 = polluted; 0 = not polluted).
Flag_NH_4_^+^	Flag indicating the quality of the ammonium time series (turbo pump failures rendered ammonium measurements very noisy in May and June, 1 = good; 0 = bad).
**APS**	
Size bin’s lower boundary (1.06, 1.13, …, 15.95)	Particle normalized concentrations for each bin (dNdlog/Dp), the header marks the lower bin boundary of the corrected *d*_*a*_ in µm, the uppermost boundary is 16.1 µm.
totalconc	Total particle concentration of all bins in units of particles/cm^3^.
PDA_flag	Pollution flag (1 = polluted; 0 = not polluted).
**WIBS**	
Size bin’s lower and upper boundary (0.5–0.6 µm, 0.6–0.72 µm, …, 16.63–20 µm)	Particle normalized concentrations [cm^−3^] of excited, fluorescent, and hyper-fluorescent aerosols (dNdlog/Dp) (three distinct datasets). The header marks the lower and the upper bin boundary of the corrected optical diameter in µm.
Excited_conc_[1/cm^3^]	Particle number concentration [cm^−3^] of excited aerosols.
Fluorescent_conc_[1/cm^3^], Fluorescent_A_conc_[1/cm^3^], …, Fluorescent_ABC_conc_[1/cm^3^]	Particle number concentration [cm^−3^] of fluorescent aerosols and of fluorescent aerosols of type A, B, C, AB, AC, BC and ABC.
Hyper-Fluorescent_conc_[1/cm^3^], Hyper-Fluorescent_A_conc_[1/cm^3^], …, Hyper-Fluorescent_ABC_conc_[1/cm^3^]	Particle number concentration [cm^−3^] of hyper-fluorescent aerosols and of hyper-fluorescent aerosols of type A, B, C, AB, AC, BC and ABC.
**CCNC**	
Concentration_[1/cm^3^]	Number concentration of CCN in units of CCN/cm^3^, for the supersaturation levels of 0.15, 0.2, 0.3, 0.5, and 1%.
PDA_flag	Pollution flag (1 = polluted; 0 = not polluted).
**CPC3025 & CPC3776**	
Concentration_[1/cm^3^]	Corrected particle number concentration from the interstitial (CPC3025) and total (CPC3776) inlet in units of particles/cm^3^.
Correction_flag	Inlet switching correction flag (1 = corrected; 0 = not corrected).
**Hygrometer interstitial inlet & total inlet**	
Valve_position	Switching valve position (1 = total; 0 = interstitial).
RH_[%]	Relative humidity inside interstitial/total inlet.
T_[°C]	Temperature inside interstitial/total inlet in units of °C.
**Pollution flags (PDA applied on CPC3025 & CPC3776 datasets)**	
Concentration_[1/cm^3^]	Corrected particle number concentration from the interstitial/total inlet in units of particles/cm^3^.
PDA_flag	Pollution flag (PDA applied on CPC3025/CPC3776 datasets, 1 = polluted; 0 = not polluted).

The ARM CCNC, SMPS, and UHSAS datasets are freely available on the ARM open access data repository (https://adc.arm.gov/discovery/#/).

## Technical Validation

### AMS vs. SMPS mass closure

A mass closure analysis was performed between the total NR-PM_1_ calculated from the AMS (Eq. 2) and the one approximated from the particle counts, at mobility diameters (*d*_*m*_), measured by the SMPS located in the ARM container. We subtracted the aethalometer-derived eBC mass from the SMPS-derived PM_1_ mass, since BC is not measured by the AMS due to its refractory nature.

2 $$\begin{array}{c}{\left[NRPM1\right]}_{AMS}=\left[N{O}_{3}^{-}\right]+\left[S{O}_{4}^{2-}\right]+\left[N{H}_{4}^{+}\right]+\left[C{l}^{-}\right]+\left[Org\right]\end{array}$$

To account for the difference in cutoff sizes between the AMS (<1 µm, *d*_*va*_) and the SMPS (10–500 nm, *d*_*m*_), we assumed that the particles’ shape is spherical, and it follows that *d*_*va*_ is roughly equal to *d*_*m*_, multiplied by the time and chemical-dependant particle density (*ρ*_*comp*_, Eq. 3)^70–73^.

3 $$\begin{array}{c}{\rho }_{comp}=\frac{\left[N{O}_{3}^{-}\right]+\left[S{O}_{4}^{2-}\right]+\left[N{H}_{4}^{+}\right]+\left[C{l}^{-}\right]+\left[eBC\right]+\left[Org\right]}{\frac{\left[N{O}_{3}^{-}\right]+\left[S{O}_{4}^{2-}\right]+\left[N{H}_{4}^{+}\right]}{1.75}+\frac{\left[C{l}^{-}\right]}{1.52}+\frac{\left[eBC\right]}{1.77}+\frac{\left[Org\right]}{1.2}}\end{array}$$

We assumed a density of 1.75 g/cm^3^ for ammonium nitrate and ammonium sulfate^74^, 1.52 g/cm^3^ for ammonium chloride^74^, 1.2 g/cm^3^ for organic aerosols^75^, and 1.77 g/cm^3^ for black carbon^76^. When translated into *d*_*va*_, SMPS measurements ranged from 13 to 600 nm. We then compared the AMS mass integrated over all sizes versus the mass integrated between 13 and 600 nm in *d*_*va*_, and retrieved the slope of the linear regression between the two. The difference in cutoff sizes between the two instruments was then accounted for by dividing the AMS mass by the resulting slope value of 1.2622. The SMPS-derived PM_1_ mass was then calculated using *ρ*_*comp*_ and assuming spherical particle shape. Both AMS and SMPS-derived PM_1_ time series were averaged to the common resolution of 10 min (that of the final eBC dataset) before proceeding to the comparison.

The mass closure analysis was performed independently for the three periods Oct-Dec, Mar-May and Jun-Jul, and yielded the following slopes (1/scaling factors): 0.271 (R^2^ = 0.016), 1.543 (R^2^ = 0.816) and 2.893 (R^2^ = 0.912) for the three respective periods, as shown in Fig. 3. Note that all datapoints under the influence of local pollution emissions were excluded from the analysis. Furthermore, the period from November 10^th^ to December 5^th^, 2019 was also excluded from the Oct-Dec mass closure analysis, because of nearly continuous storm conditions, which resulted in strong discrepancies between the AMS and SMPS (potentially related to elevated sea salt concentration that could not be measured by the AMS due to their refractory nature). Hence, it is worth mentioning that sea salt is missing in this mass closure analysis and therefore the scaled AMS results represent an upper estimate. Finally, the scaling factors are not applied by default on the final dataset and are given as a reference depending on the needs of the user. We acknowledge that there is a large variability in the range of scaling factors depending on the period of the year, and overall large discrepancies between the AMS-derived PM_1_ and the SMPS-derived one. Nevertheless, as discussed in the “Aerosol chemical composition and mass concentration” section, the AMS measurement uncertainties are subject to a variety of influencing factors, including the estimation of *IE*_*NO*3_ and *RIE*s from calibrations, the calculation of the CDCE and the single ion counting errors, that may add up to nearly 40% overall uncertainty^62,77^. These uncertainties are also much larger than the range of calculated inlet losses for the AMS sampling line (mostly below 10%, as presented in the “Inlet losses characterization” section) so that the losses cannot serve as an argument to explain the observed discrepancies. The fact that the instrument was each time in a different state, after long down times, can also explain part of the variability observed here. Also, the sea salt contribution to the aerosol population changes throughout the year^22^ and introduces various degrees of PM_1_ mass underestimation. Despite these discrepancies, the SMPS and AMS signals co-vary similarly, as inferred from the high R^2^ value for Mar-May (R^2^ = 0.816) and Jun-Jul (R^2^ = 0.912) periods (less so for the Oct-Dec period, where the forcing of the regression through the origin introduces a large bias). Given (1) the relative measurement uncertainties for both instruments (about 10% for the SMPS^78^ and up to 40% for the AMS^62^), (2) the fundamentally different measurement methods employed by the two instruments, and (3) the low aerosol mass concentration in the Arctic that makes the instruments work close to their detection limits, we believe that the measurements performed with the AMS are trustworthy, especially when the scaling factors calculated here are applied to the dataset.

#### Comparison of particle number size distribution from the APS and WIBS

To compare the size distribution of the APS and WIBS, we first corrected the APS aerodynamic diameter *d*_*a*_ based on experiments with Polystyrene latex (PSL) spheres (Eq. 4).

4 $$\begin{array}{c}{d}_{acorrected}=0.8\cdot {d}_{a}+0.1\end{array}$$

Then, *d*_*a corrected*_ was converted to the physical diameter *d*_*p*_, assuming it is equivalent to the optical diameter (Eq. 5)^72^.

5 $$\begin{array}{c}{d}_{p}=\frac{{\rho }_{0}}{{\rho }_{p}}{d}_{a}\chi \end{array}$$

**Fig. 4:**
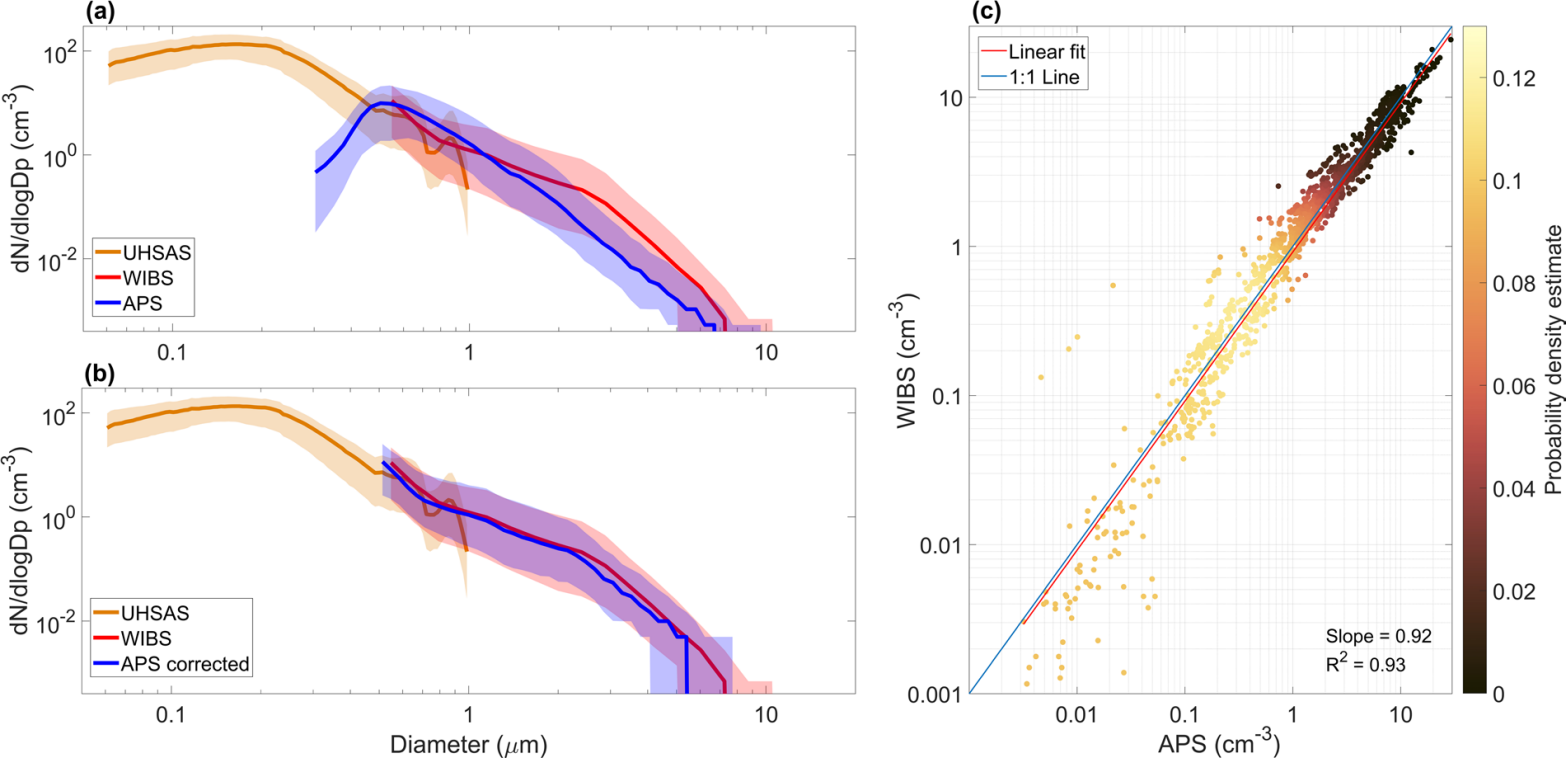
Comparison of the particle size distributions from the APS, WIBS and USHAS (**a**) before correction and (**b**) after correction of the APS aerodynamic diameter. For (**a**) and (**b**), the thick lines represent the median dN/dlogDp at each size bins for the entire expedition, where polluted data were removed, and the shaded envelops represent the corresponding 25 and 75% quantiles. The comparison of the APS and WIBS total concentration after correction is shown in panel (**c**), where the reader can refer to the caption in Fig. 3 for a description of the color bar.

Where *ρ*_*p*_ = 2.17 g cm^−3^ is the particle density based on the assumption that sea salt (NaCl) is the most abundant contribution to the central Arctic coarse mode aerosol, *ρ*_*0*_ = 1 g cm^−3^ is the standard density, and *χ* is the particle shape factor, which was set to 1.05 based on Zieger *et al*.^79^. The formula is typically used for smaller particle sizes for which the free molecular regime applies^72^ but resulted in the best agreement of the overlapping diameters in the particle number size distributions of the APS and WIBS. The APS number size distributions are only reported for diameters larger than 1.058 μm (*d*_*a*_) as the concentration drop for smaller sizes disagrees with the WIBS and UHSAS data (Fig. 4b). After correction, a good agreement was found between the APS and the WIBS total concentration (R^2^ = 0.93, Fig. 4c).

#### CCN number closure and comparison with the ARM CCN dataset

**Fig. 5:**
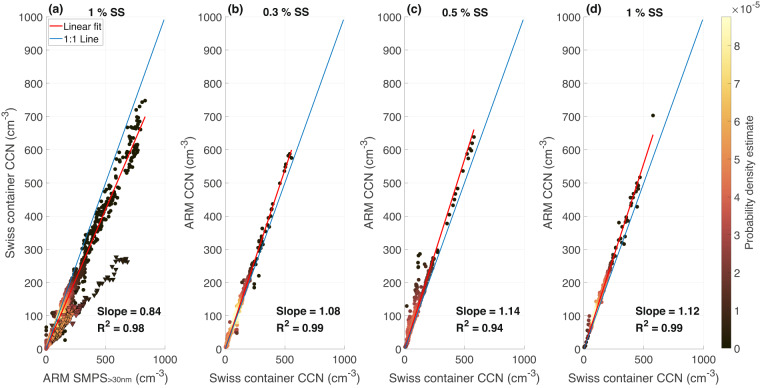
Number closure analysis between the CCNC and SMPS_>30nm_ (**a**) and comparison with the ARM CCNC at different SS (**b**–**d**). Points with a triangular shape and black edges in (**a**) correspond to a period of very high eBC concentration (as measured with the aethalometer) between December 5^th^ and December 12^th^ 2019, that are excluded in the computation of the linear regression fit. The number of data points available for comparison between the Swiss container CCNC and the ARM CCNC (**b**–**d**) is small, because periods where both instruments measured at the same SS level are limited. 532, 247, and 314 data points were used for the comparison at SS 0.3, 0.5, and 1%, respectively. Refer to the caption in Fig. 3 for a description of the color bar.

To validate the measurements performed with the CCNC, we first performed a number-closure study with the SMPS data. At very high supersaturations (SS), most particles activate as droplets and the CCN concentrations measured by the CCNC are expected to be comparable, under certain conditions, to the total particle number concentrations derived from the size distribution measurements of the SMPS^46^. In practice, we expect the CCN number concentrations measured with the CCNC at 1% SS to be equal to the integrated SMPS number concentrations, starting with a diameter larger than 30 nm (SMPS_>30nm_, particles smaller than 30 nm might be below the activation diameter and will not be counted by the CCNC)^46^. The results from this analysis are presented in Fig. 5a, where the slope of the linear regression between the particle number concentrations measured with the CCNC at 1% SS versus the SMPS_>30nm_ is equal to 0.84 (R^2^ = 0.98). Note that a period between December 5^th^ and 12^th^ 2019 was excluded from the regression computation, as elevated concentrations of (hydrophobic/non-hygroscopic) eBC (as measured with the aethalometer) greatly influenced the particles’ droplet activation and thus the CCN count from the CCNC. We also compared the measurement from our CCNC with those from the neighbouring CCNC in the ARM container, at 0.3, 0.5 and 1% SS (Fig. 5b–d). The two instruments agree well in general, with differences ranging from 8% at 0.3% SS to 14% at 0.5% SS, which fall within the range of uncertainty for both instruments (≈ 10%, propagated to 22.5% when considering the intercomparison of the two^46,63^).

#### Intercomparison between the two CPCs

**Fig. 6:**
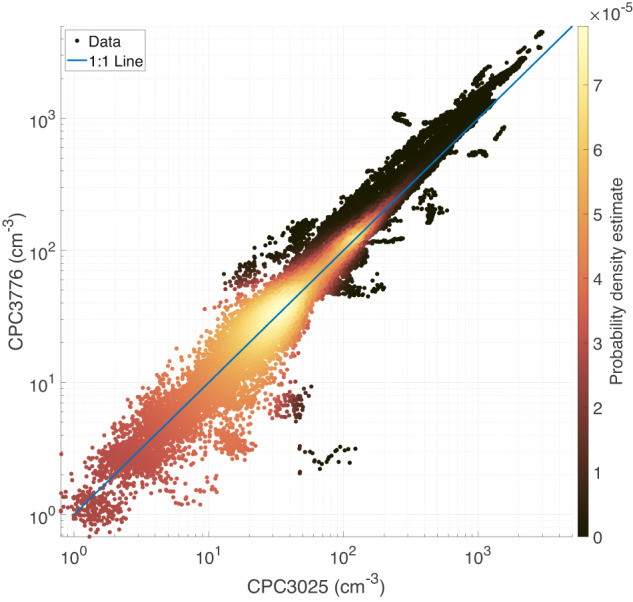
Comparison of the two CPCs. Note that both axes are on a logarithmic scale to cover the wide range of particle number concentrations. The fitted linear regression (not shown because of the logarithmic axes) between the two instrument has a slope of 1.166 (R^2^ = 0.95). Refer to the caption in Fig. 3 for a description of the color bar. Some data points deviate from the 1:1 line on the 15^th^ of November (bottom right of the point cloud) due to polluted points detected by the CPC3025 and not by the CPC3776 that failed to be identified as such by the PDA.

The particle number concentrations measured by the two CPCs were compared to assure the quality of each dataset. For the comparison, datapoints under the influence of local pollution from ship emissions were discarded and 1 min averaged data were used for both CPCs. Figure 6 shows the results of this comparison, where a slope of 1.166 (R^2^ = 0.95) was found for the fitted linear regression between the CPC3776 and CPC3025 measured number concentrations. In general, the measurements from the two instruments agree well, with a tendency for the CPC behind the interstitial inlet (CPC3025) to underestimate particle number concentrations during periods of high number concentrations for typical Arctic air (approx. > 300 particles/cm^3^). These periods were mainly observed during summer months (June, July and August) where the particle size distribution is dominated by ultrafine and Aitken mode particles^30^, with a number of NPF events. As the minimum nominal detectable particle diameter differs for the two instruments (2.5 nm for the CPC3776 and 3 nm for the CPC3025), the occurrence of NPF events contributes to the observed difference at high particle number concentrations between the two instruments (not shown).

## Usage Notes

We strongly encourage the reader to refer to the datasets’ metadata, published on PANGAEA (see Sect. Data Records), for further details on data usage. Pollution flags and/or quality-check flags are provided with each dataset and should be carefully applied before further data analysis. For the AMS, the scaling factors from the mass closure analysis with the SMPS are not applied to the dataset by default. The user is left with the decision to apply them or compute new ones.

The temperature measurements in the total and interstitial inlet can be used to convert measurements into Standard Temperature and Pressure (STP) conditions.

## Data Availability

The pollution detection algorithm described in Beck *et al*.^80^ to identify and flag periods of primary polluted data is available on Zenodo (10.5281/zenodo.5761101). High Resolution ToF-AMS Analysis guide from J. L. Jimenez research group’s wiki (CIRES, University of Colorado at Boulder, USA): https://cires1.colorado.edu/jimenez-group/wiki/index.php/High_Resolution_ToF-AMS_Analysis_Guide (last accessed: 03/03/2022).
